# A novel mutation in LRSAM1 causes axonal Charcot-Marie-Tooth disease with dominant inheritance

**DOI:** 10.1186/1471-2377-14-118

**Published:** 2014-06-03

**Authors:** Maik Engeholm, Julia Sekler, David C Schöndorf, Vineet Arora, Jens Schittenhelm, Saskia Biskup, Caroline Schell, Thomas Gasser

**Affiliations:** 1 Department of Neurology and Hertie Institute for Clinical Brain Research, Hoppe-Seyler-Str. 3, 72076 Tübingen, Germany; 2 German Center for Neurodegenerative Diseases (DZNE), Otfried-Müller-Str. 27, 72076 Tübingen, Germany; 3 Graduate School of Cellular & Molecular Neuroscience, Österbegstr. 3, 72074 Tübingen, Germany; 4 Division of Neuropathology, Calwerstr. 3, 72076 Tübingen, Germany; 5 CeGaT GmbH, Paul-Ehrlich-Str. 17, 72076 Tübingen, Germany

**Keywords:** Axonal CMT, LRSAM1, Anterior horn cell disease, Splice site mutation, RING domain, Exome sequencing

## Abstract

**Background:**

Charcot-Marie-Tooth disease (CMT) refers to a heterogeneous group of genetic motor and sensory neuropathies. According to the primary site of damage, a distinction is made between demyelinating and axonal forms (CMT1 and 2, respectively, when inherited as an autosomal dominant trait). Leucine-rich repeat and sterile alpha motif-containing protein 1 (LRSAM1) is a ubiquitin-protein ligase with a role in sorting internalised cell-surface receptor proteins. So far, mutations in the LRSAM1 gene have been shown to cause axonal CMT in three different families and can confer either dominant or recessive transmission of the disease.

**Case presentation:**

We have identified a novel mutation in LRSAM1 in a small family with dominant axonal CMT. Electrophysiological studies show evidence of a sensory axonal neuropathy and are interesting in so far as giant motor unit action potentials (MUAPs) are present on needle electromyography (EMG), while motor nerve conduction studies including compound motor action potential (CMAP) amplitudes are completely normal. The underlying mutation c.2046+1G >T results in the loss of a splice donor site and the inclusion of 63 additional base pairs of intronic DNA into the aberrantly spliced transcript. This disrupts the catalytically active RING (Really Interesting New Gene) domain of LRSAM1.

**Conclusions:**

Our findings suggest that, beyond the typical length-dependent degeneration of motor axons, damage of cell bodies in the anterior horn might play a role in LRSAM1-associated neuropathies. Moreover, in conjunction with other data in the literature, our results support a model, by which disruption of the C-terminal RING domain confers dominant negative properties to LRSAM1.

## Background

CMT comprises a clinically and genetically heterogeneous group of inherited motor and sensory neuropathies [1]. With an overall prevalence of approximately 1 in 2,500 individuals, CMT is the most common genetic disorder of the PNS [2]. In a majority of cases, CMT is transmitted as an autosomal dominant trait and is further classified as CMT1 or CMT2 according to the primary site of damage (demyelinating and axonal, respectively) [1,3]. In other families, CMT is transmitted as an X-linked or autosomal recessive trait. These latter cases are commonly classified as CMT4 when they show a demyelinating phenotype, while axonal forms are referred to as autosomal recessive CMT2 (AR-CMT2) [4]. Since the identification of a duplication of the peripheral myelin protein 22 locus as the cause of CMT1A more than 20 years ago [5,6], mutations in more than 60 genes implicated in a variety of different cellular functions have been associated with various forms of CMT [3]. Beyond their relevance for clinical classification, these genes identify molecules and molecular pathways that play a primary role for the structural and functional integrity of PNS neurons and their myelin sheath, respectively, and are potential targets for future therapeutic interventions.

Three different mutations in the LRSAM1 gene have been shown to cause axonal CMT in humans. Guernsey et al. [7] reported a large, multiply consanguineous family from Eastern Canada, in which axonal CMT was inherited as an autosomal recessive trait. The clinical presentation included moderate weakness and wasting, predominantly affecting distal lower limb muscles, with an onset in early adulthood. Needle EMG revealed signs of denervation and reinnervation, and sensory nerve action potentials (SNAPs) were reduced or absent. Homozygosity mapping yielded a splice site mutation c.1913-1G >A in the LRSAM1 gene giving rise to a premature stop codon 20 bp inside the penultimate exon. Subsequently, two further mutations in LRSAM1 were identified in a Dutch and a Sardinian family with dominant axonal CMT [8,9]. In both studies, the clinical and electrophysiological findings were very similar to that reported by Guernsey et al. [7]. Both mutations, p.Leu708ArgfsX28 and p.Ala683ProfsX3, disrupt the RING domain of LRSAM1.

**Figure 1:**
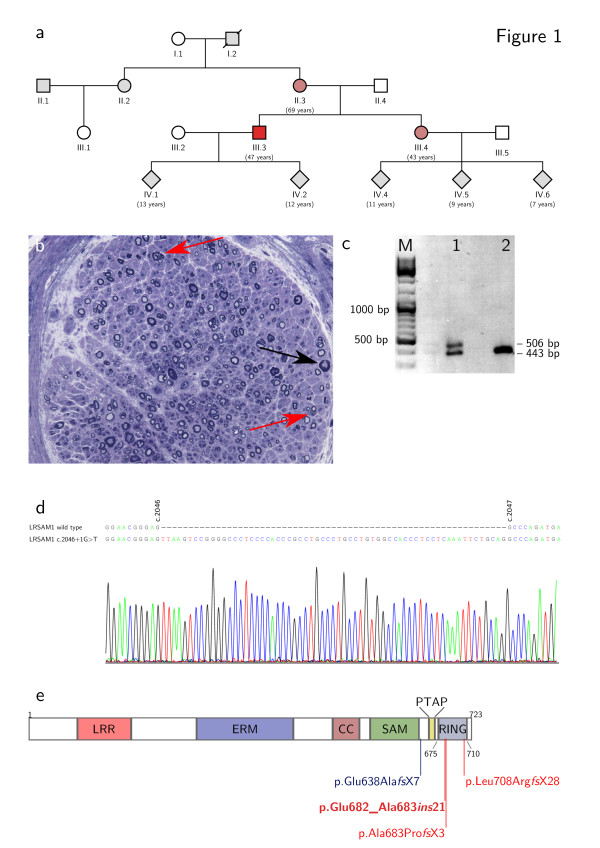
**Pedigree, biopsy and molecular biology studies. a)** Pedigree of the family. The index patient is individual III.3. **b)** Tolouidine blue-stained semi-thin section of a sural nerve biopsy from the index patient. Shown are several small clusters of axons as an indication of axonal regeneration (red arrows) and one axon undergoing acute Wallerian degeneration (black arrow). The overall number of axons is slightly reduced. **c)** RT-PCR amplification of LRSAM1 mRNA from blood of the index patient (lane 1) and a healthy control patient (lane 2). **d)** Sanger sequencing of the PCR product from **c**. Shown is a sequence alignment of the wild type sequence (top) and the sequence obtained from the larger size PCR product in the index patient (bottom and chromatogram). **e)** LRSAM1 domain structure and sites of disease causing mutations. LRR - leucine rich repeat domain; ERM - ezrin-radixin-moezin domain; CC - coiled coil region; SAM - sterile alpha motif domain; PTAB - PTAB motif domain; RING - RING finger domain. Positions are indicated by amino acid number. The recessive mutation from Guernsey et al. is highlighted blue, dominant mutations are highlighted red, the mutation reported herein is in bold and dark red.

LRSAM1 is a E3 ubiquitin-protein ligase highly conserved throughout vertebrate evolution [10]. Alternative splicing gives rise to three different isoforms in humans, the largest of which consists of 723 amino acids and harbours an N-terminal leucine-rich repeat domain, an ezrin-radixin-moezin domain, a coiled-coil region, a sterile alpha motif domain and a C-terminal C3HC4-type RING finger domain (Figure 1e). In human and mouse, LRSAM1 is highly expressed in motor neurons of the spinal cord and cell bodies of sensory neurons of dorsal root ganglia [8,11]. Moreover, some expression is observed in the central nervous system [10,11]. In a cell culture system, LRSAM1 has been shown to interact with and mediate monoubiquitination of the Tumour susceptibility gene 101 protein (TSG101) [10]. TSG101 is a component of the ESCRT (Endosomal Sorting Complexes Required for Transport)-1 complex, which is involved in the sorting of endocytic ubiquitinated cargoes into lumenal vesicles of late endosomes [12]. Upon LRSAM1-mediated ubiquitination, TSG101 relocalises from these multivesicular bodies to a detergent-soluble compartment and loses its ability to direct internalised receptor proteins to the lysosome [10]. In a mouse model, loss of LRSAM1 results in decreased motor NCVs and motor axon counts following acrylamide challenge, while neuromuscular function is otherwise unimpaired [11].

Here, we report a novel mutation in LRSAM1 in a small family with autosomal dominant axonal CMT. We present an in-depth electrophysiological examination of three affected individuals and characterise the functional consequences of the splice site mutation at the level of the transcript. Our results provide new insights into the pathomechanism of LRSAM1-associated neuropathies, both at the cellular and molecular level.

## Case presentation

We present a family with three members suffering from a predominantly sensory neuropathy with fasciculations and hyper-CK-aemia but without evidence of motor axonal neuropathy in nerve conduction studies (Figure 1a). The 47-years old index patient complained of a reduced sensation for touch on his feet, for instance when trying on shoes, which he had first noticed more than 20 years ago. Over the past few years, these deficits had gradually spread over his lower legs and recently also reached his finger tips. For several years he had noticed an unsteady gait, especially when walking in the dark, and he reported long-standing fasciculations of his calf and thigh muscles as well as crampi affecting both the leg and hand muscles. Over the past year, he had observed a reduction in size of his calf muscles. He did not complain of weakness, paresthesias or vegetative symptoms. His 43-years old sister and his 69-years old mother had very similar complaints with an age of onset between 20 and 40 years.

Clinical examination of the index patient showed fasciculations of proximal leg muscles. The intrinsic foot and hand muscles appeared slender, but no other signs of atrophy or skeletal deformities were observed. There was no motor weakness. Deep tendon reflexes were normal in the upper extremities but absent in the lower extremities. Sensation for touch, vibration, heat and pain was markedly reduced in the lower legs, and tandem gait was mildly impaired. While clinical examination of the sister gave very similar results, in the mother an additional grade 4/5 weakness of ankle dorsiflexion and intrinsic hand muscles was present. Laboratory testing revealed a moderate hyper-CK-aemia in all three individuals (maximum 480 U/l in the mother), while other parameters were normal. Electrodiagnostic studies yielded similar findings in all three individuals (Table 1). Firstly, sensory nerve conduction studies showed reduced SNAPs in upper and lower extremity nerves. Secondly, while there was no evidence of acute denervation on needle examination, giant MUAPs were observed both in proximal and distal muscles. Thirdly, motor nerve conduction studies including CMAPs were completely normal. A biopsy of the sural nerve in the index patient showed a chronic neuropathy with predominantly axonal damage (Figure 1b).

**Table 1:** Electrophysiological studies

	**Index**		**Sister**		**Mother**	
**Nerve**	**CMAP/mV**	**mNCV/ ms**^**-1**^	**CMAP/mV**	**mNCV/ ms**^**-1**^	**CMAP/mV**	**mNCV/ ms**^**-1**^
N. ulnaris [4.0 mV / 50 ms^-1^]	23	68	19	61	15	57
N. tibialis [5.0 mV / 40 ms^-1^]	11	51	24	51	6.4	40
N. peronaeus [4.0 mV / 41 ms^-1^]	9.9	47	n.d.	n.d.	n.d.	n.d.
	**Index**		**Sister**		**Mother**	
**Nerve**	**SNAP/ *μ *** **V**	**sNCV/ ms**^**-1**^	**SNAP/ *μ *** **V**	**sNCV/ ms**^**-1**^	**SNAP/ *μ *** **V**	**sNCV/ ms**^**-1**^
N. radialis [16 *μ*V / 55 ms^-1^]	**8.8**	67	**15**	55	**6.1**	58
N. suralis [3.8 *μ*V / 39 ms^-1^]	**1.6**	50	**2.7**	43	**Ø**	n.a.
	**Index**		**Sister**		**Mother**	
**Muscle**	**ASA**	**MUAP/mV**	**ASA**	**MUAP/mV**	**ASA**	**MUAP/mV**
M. deltoideus [1.5 mV]	Ø	**2.0**	Ø	**2.7**	Ø	**6.4**
M. IOD1 [2.3 mV]	Ø	**7.5**	n.d.	n.d.	Ø	**12**
M. vastus medialis [2.0 mV]	Ø	**16**	Ø	**28**	Ø	**28**
M. tibialis anterior [1.6 mV]	Ø	**4.3**	n.d.	n.d.	n.d.	n.d.

Motor nerve conduction studies, sensory nerve conduction studies and needle EMG recorded from the index patient (index), his sister and his mother. CMAP - compound motor action potential; mNCV - motor nerve conduction velocity; SNAP - sensory nerve action potential; sNCV - sensory nerve conduction velocity; M. IOD1 - musculus interosseus dorsalis 1; ASA - abnormal spontaneous activity (fibrillation potentials or positive sharp waves); Ø - not present; n.d. - not determined; n.a. - not applicable. Normal ranges for CMAP, SNAP and MUAP amplitudes as well as NCVs are indicated in brackets in column 1. Pathological results are highlighted bold.

Genetic testing was performed on the index patient by means of whole exome sequencing. Data analysis was confined to a group of 46 candidate genes, which had previously been associated with CMT (a complete list is available on request). Only variants in these genes that had an allele frequency of less than 0.05 and could not be classified as benign based on current knowledge were considered further. This revealed a single heterozygous mutation in LRSAM1 c.2046+1G >T, which could be confirmed by Sanger sequencing and was also present in the sister and mother. Other family members were not available for testing. The mutation destroys the splice donor site downstream of exon 25. To test if the mutant allele is expressed and to map the alternative splice donor site, RT-PCR was performed on RNA isolated from blood using primers located in exon 23 and 26 (LRSAM1_c2424_fw: acctgctgagccaaatgagc and LRSAM1_c2803_rv: tcagctgctgtggtagatgc). In a healthy control subject, this yielded a single product of 443 bp, as expected. In the index patient, however, a second slightly larger band was observed (Figure 1c). Sanger sequencing showed that this second band corresponds to an aberrantly spliced LRSAM1 transcript from the mutant allele, in which an alternative splice donor site 63 bp downstream of the mutated site is used (Figure 1d). This results in the insertion of 21 additional amino acids into the LRSAM1 protein leading to a disruption of the RING domain (Figure 1e and Table 2). This mutation is predicted to be deleterious by PROVEAN [13] with high confidence (PROVEAN score of -19.2).

**Table 2:** Comparison of LRSAM1 mutations

**DNA**	**Protein**	**Expression**	**RING domain**	**Transmission**	**Reference**
c.1913-1G>A	p.Glu638Ala*fs*X7	Ø	n.a.	Recessive	Guernsey et al. 2010 [7]
c.2122_2123*ins*GC	p.Leu708ArgfsX28	n.d.	Disrupted	Dominant	Weterman et al. 2012 [8]
c.2047-1G>A	p.Ala683Pro*fs*X3	+	Disrupted	Dominant	Nicolaou et al. 2013 [9]
c.2046+1G>T	p.Glu682_Ala683ins21	n.d.	Disrupted	Dominant	Herein

Overview of mutations in LRSAM1 associated with axonal CMT. Expression refers to protein expression as determined by Western blotting in whole-cell lysates of patient-derived lymphoblastoid cell lines. The RING domain (amino acid 675-710) is called disrupted when not present in its entirety.

## Conclusions

We report a small family with autosomal dominant axonal CMT associated with a novel mutation in LRSAM1. In the affected individuals sensory deficits including impaired sensation of touch and disturbed proprioception dominate the clinical picture. Motor symptoms are present as well but are limited to fasciculations and crampi in the younger two individuals, while only the 70-years old mother showed distal predominant weakness. The onset of symptoms was reported between the second and fourth decade in all individuals, and the clinical course is relatively mild and benign. The underlying mutation in the LRSAM1 gene destroys the splice donor site at the 3’-end of exon 25. Instead, an alternative splice site 63 bp further downstream is used. This results in the insertion of 21 amino acids into the RING domain of LRSAM1 most likely rendering this domain catalytically inactive (Table 2). All in all, the clinical features present in our patients are very similar to those reported for the three other families. CMT associated with mutations in LRSAM1 is now referred to as CMT2P, irrespective of its (dominant or recessive) mode of inheritance [1,3].

We have observed rather distinctive electrodiagnostic findings in our family, which in some respect differ from those generally reported in patients with axonal CMT. Despite clear motor involvement on clinical and laboratory examination, including distal predominant weakness in the mother and fasciculations and crampi in all three individuals, motor nerve conduction studies (including CMAP amplitudes) were entirely normal (Table 1). At the same time, needle EMG showed giant MUAPs both in proximal and distal muscles of all individuals. Such giant potentials are frequently observed in slowly progressive forms of anterior horn cell disease. The finding might be taken as an indication that in the patients reported here cellular damage affects not only the axon in a typical length-dependent manner but also the cell body of the lower motor neuron. Consistent with this idea, denervation of paraspinal muscles has been documented in one family with LRSAM1-associated CMT [7]. The normal CMAP amplitudes, similar to giant MUAPs, may be considered the result of extensive axonal regeneration in LRSAM1-associated neuropathies. However, all in all, further detailed reports of electrophysiological and histopathological findings in genetically defined neuropathies are needed to address the question of how well this type of study can provide information regarding the subcellular site of damage or the kinetics of degenerative and regenerative processes.

An interesting feature of LRSAM1 is that different mutations in the same gene cause either recessive or dominant forms of CMT. A similar behaviour has been observed for a number of other disease-associated genes, and the underlying molecular mechanisms are subject of intensive research [14-17]. In the case of LRSAM1, the mutation described by Guernsey et al. [7] is likely to be a null mutation due to a stop codon in the penultimate exon of the aberrantly spliced transcript. The stop codon is normally found within the very last exon of a gene, and its premature occurrence in any other exon can trigger degradation of the mRNA by a mechanism referred to as nonsense-mediated decay [18]. Consistent with this notion, Guernsey et al. [7] have failed to detect LRSAM1 protein in blood cells of homozygous mutation carriers (Table 2).

The absence of clinical symptoms in heterozygous carriers of the null mutation described by Guernsey et al. [7] suggests that the other three mutations reported by Weterman et al. [8], Nicolaou et al. [9] and herein exert a dominant negative effect, rather than lead to haploinsufficiency. Consistent with this idea, the mutant protein could be detected in blood cells of heterozygous mutation carriers in one family [9]. It is tempting to speculate that such a dominant negative effect results immediately from the disruption of the RING domain, where all three mutations occur (Table 2). Artificially engineered mutations inactivating the RING domain of LRSAM1 not only fail to confer ubiquitination, but at the same time show an increased physical association with the substrate TSG101 [10]. This appears to reinforce the endosomal localisation of TSG101 and enhance its sorting activity, possibly through blocking its ubiquitination by wild-type LRSAM1 or other ubiquitin ligases. It is conceivable that a similar type of blocking interaction occurs between catalytically inactive mutants of LRSAM1 and other physiological substrates.

Further studies are needed to investigate if the misregulation of ESCRT complexes in LRSAM1 mutation carriers is responsible for the CMT phenotype or if other yet unknown functions of LRSAM1 also play a role. In this context, it is worth mentioning that the product of another CMT2-associated gene, small integral membrane protein of lysosome/late endosome (SIMPLE), also interacts with various components of ESCRT complexes including TSG101 [19]. Note however, that in cell culture experiments SIMPLE and LRSAM1 (as well as the respective disease-associated mutations) have opposite effects on the function of ESCRT complexes: While SIMPLE promotes the assembly of endosomal ESCRT complexes and thereby accelerates lysosomal degradation of activated growth factor receptors, LRSAM1-dependent ubiquitination causes the disassembly of these sorting complexes resulting in prolonged signalling of the activated receptors [10,20]. It is conceivable that a tightly controlled balance between the two processes is a prerequisite for an optimal regulation of neurotrophic signalling in PNS neurons. The use of patient-derived induced pluripotent stem cells [21], which can be differentiated into motor neurons [22], will offer the opportunity to study these processes in a more authentic context than previous cell culture experiments.

Finally, our study illustrates the power of targeted exome analyses as a diagnostic procedure especially for patients with neurodegenerative disorders. As a first step, the approach involves whole exome sequencing using standard next-generation sequencing technologies. Subsequent data analysis, however, is confined to genes that have previously been associated with the clinically suspected diagnosis. In our case, this group comprised 46 genes at the time of examination. In our experience, this strategy is far more efficient than are conventional sequencing panels or the successive sequencing of individual candidate genes, especially when dealing with genetically diverse diseases such as CMT [1,3]. In addition, it provides a high degree of flexibility, as it is possible to re-assess the original exome data at a later stage in case new genes are added to a list of candidate genes or new symptoms appear in the course of a disease indicating a different clinical diagnosis. Our case illustrates that by this method it is possible to identify novel heterozygous disease causing mutations, as long as they occur within a gene that belongs to a suitable group of candidate genes defined by clinical considerations.

## Consent

Written informed consent was obtained from the patient for publication of this Case report and any accompanying images. A copy of the written consent is available for review by the Editor of this journal.

## Abbreviations

CMAP: Compound motor action potentials; CMT: Charcot-Marie-Tooth disease; EMG: Electromyography; ESCRT: Endosomal sorting complexes required for transport; LRSAM1: Leucine-rich repeat and sterile alpha motif-containing protein 1; MUAP: Motor unit action potentials; NCV: Nerve conduction velocities; PNS: Peripheral nervous system; RING: Really interesting new gene; SIMPLE: Small integral membrane protein of lysosome/late endosome; SNAP: Sensory nerve action potential; TSG101: Tumour susceptibility gene 101 protein.

## Competing interests

The authors declare that they have no competing interests.

## Authors’ contributions

ME, CS, SB and TG saw the patient and diagnosed CMT; CS performed electrophysiological studies; JSch performed histopathological studies; SB performed exome sequencing; JS, DCS, VA, ME and TG performed molecular biology studies. ME wrote the manuscript with contributions from all other authors. All authors read and approved the final version of the manuscript.

## Pre-publication history

The pre-publication history for this paper can be accessed here:

http://www.biomedcentral.com/1471-2377/14/118/prepub
